# Rheumatoid arthritis–associated autoantibodies in non–rheumatoid arthritis patients with mucosal inflammation: a case–control study

**DOI:** 10.1186/s13075-015-0690-6

**Published:** 2015-07-09

**Authors:** Koen M J Janssen, Menke J de Smit, Elisabeth Brouwer, Fenne A C de Kok, Jan Kraan, Josje Altenburg, Marije K Verheul, Leendert A Trouw, Arie Jan van Winkelhoff, Arjan Vissink, Johanna Westra

**Affiliations:** Department of Oral and Maxillofacial Surgery, University of Groningen and University Medical Center Groningen, Groningen, The Netherlands; Center for Dentistry and Oral Hygiene, University of Groningen and University Medical Center Groningen, Groningen, The Netherlands; Department of Rheumatology and Clinical Immunology, University of Groningen and University Medical Center Groningen, PO Box 30.001, 9700 RB Groningen, The Netherlands; Department of Pulmonology, University of Groningen and University Medical Center Groningen, Groningen, The Netherlands; Department of Pulmonary Diseases, Medical Center Alkmaar, Alkmaar, The Netherlands; Department of Rheumatology, Leiden University Medical Center, Leiden, The Netherlands; Department of Medical Microbiology, University of Groningen and University Medical Center Groningen, Groningen, The Netherlands

## Abstract

**Introduction:**

Rheumatoid arthritis–associated autoantibodies (RA-AAB) can be present in serum years before clinical onset of rheumatoid arthritis (RA). It has been hypothesized that initiation of RA-AAB generation occurs at inflamed mucosal surfaces, such as in the oral cavity or lungs. The aim of this study was to assess systemic presence of RA-AAB in patients without RA who had oral or lung mucosal inflammation.

**Methods:**

The presence of RA-AAB (immunoglobulin A [IgA] and IgG anti-cyclic citrullinated peptide 2 antibodies (anti-CCP2), IgM and IgA rheumatoid factor (RF), IgG anti-carbamylated protein antibodies and IgG and IgA anti-citrullinated peptide antibodies against fibrinogen, vimentin and enolase) were determined in sera of non-RA patients with periodontitis (PD, n = 114), bronchiectasis (BR, n = 80) or cystic fibrosis (CF, n = 41). Serum RA-AAB levels were compared with those of periodontally healthy controls (n = 36). Patients with established RA (n = 86) served as a reference group. Association of the diseases with RA-AAB seropositivity was assessed with a logistic regression model, adjusted for age, sex and smoking.

**Results:**

Logistic regression analysis revealed that IgG anti-CCP seropositivity was associated with BR and RA, whereas the association with PD was borderline significant. IgA anti-CCP seropositivity was associated with CF and RA. IgM RF seropositivity was associated with RA, whereas the association with BR was borderline significant. IgA RF seropositivity was associated with CF and RA. Apart from an influence of smoking on IgA RF in patients with RA, there was no influence of age, sex or smoking on the association of RA-AAB seropositivity with the diseases. Anti-CarP levels were increased only in patients with RA. The same held for IgG reactivity against all investigated citrullinated peptides.

**Conclusion:**

Although overall levels were low, RA-AAB seropositivity was associated with lung mucosal inflammation (BR and CF) and may be associated with oral mucosal inflammation (PD). To further determine whether mucosal inflammation functions as a site for induction of RA-AAB and precedes RA, longitudinal studies are necessary in which RA-AAB of specifically the IgA isotype should be assessed in inflamed mucosal tissues and/or in their inflammatory exudates.

## Introduction

The first autoantibody discovered in rheumatoid arthritis (RA) was rheumatoid factor (RF), which is directed against the constant domain of the immunoglobulin G (IgG) molecule. RF is not very specific for RA, as it is commonly found in other (autoimmune) diseases, too [1–4]. In contrast to RF, anti-citrullinated protein antibodies (ACPA) are highly specific (98 %) for RA [5]. ACPA can be directed against a number of citrullinated autoantigens. Production of ACPA in RA is associated with distinct genetic risk factors [6] and worse disease outcome [7].

Recently, anti-carbamylated protein antibodies (anti-CarP) were described as a third autoantibody system in RA [8]. Carbamylation is, like citrullination, a post-translational modification and results in a chemically similar structure [9]. Antibodies, however, are able to distinguish between carbamylated and citrullinated antigens. As for RF and ACPA [10, 11], the presence of anti-CarP in serum can precede clinical onset of RA and is associated independently of ACPA with a higher risk of developing RA [12].

Although the presence of ACPA and RF is of great importance in RA diagnosis, the role of these antibodies in the initiation and pathogenesis of RA has been less well elucidated. It has been hypothesized that initiation of RA-associated autoantibody (RA-AAB) generation occurs at inflamed mucosal surfaces, such as in the lung and oral mucosa [13]. IgA is the predominant antibody of the mucosal immune system, and IgA ACPA is elevated and highly specific for RA in individuals with preclinical and early RA [14–16].

Because smoking is a risk factor for RA development [17], the lungs have been speculated to play a role in RA initiation [18]. Smoking induces chronic inflammation at mucosal surfaces [19] and act as an environmental trigger for the appearance of specifically IgA ACPA before onset of RA [15]. Lung mucosal inflammation (e.g., bronchiectasis [BR]) is more commonly found in patients with RA than in the general population [20]. The capability of plasma cells in inducible bronchus-associated lymphoid tissue to produce ACPA and RF [21], as well as the increased presence of airway abnormalities in arthritis-free individuals with serum RF and/or ACPA positivity as compared with ACPA- and RF-negative controls [22], may indicate a role for the respiratory system in the initiation of RA-AAB.

The association between RA and BR suggests that RA may occur at an increased rate in patients with cystic fibrosis (CF). The prevalence of rheumatic symptoms increases with age and CF severity and is associated with lung superinfection. However, an association with definite RA is not yet established [23]. After years of antigen stimulation, episodic arthritis could progress to RA [24]. Mutations in the cystic fibrosis transmembrane conductance regulator (*CFTR*) gene may play a role in this progression [25]. Compared with healthy controls (HC), RF is increased in patients with CF, specifically in patients with CF who have episodic arthritis [24]. Up to now, there are no data on the presence of other RA-AAB in patients with CF.

As well as being a risk factor for RA, smoking is a risk factor for mucosal inflammation in the periodontal region (periodontitis [PD]) [26]. Microorganisms organized in the subgingival biofilm are the primary etiologic agents in PD. The periodontal pathogen *Porphyromonas gingivalis* has been hypothesized to be involved in ACPA initiation owing to its own peptidyl arginine deiminase (PAD) enzyme necessary for protein citrullination [27, 28]. *P. gingivalis* peptidyl arginine deiminase (PPAD) is able to citrullinate endogenous and human proteins, thereby creating antigens that have been presumed to initiate the ACPA response in RA [29]. Recently, this hypothesis has been expanded by myeloperoxidase-mediated protein carbamylation and associated anti-CarP production in the inflamed oral mucosa of patients with PD [30]. Therefore, PD has been posed as a candidate risk factor for RA [31].

Reactivity against native forms of citrullinated RA autoantigens was found in sera from patients with RA before clinical symptoms occurred [32]. Recently, reactivity against native forms of citrullinated RA autoantigens in PD patients with PD was found to be increased compared with non-PD controls [33]. These findings have raised the hypothesis that, at least in some individuals, reactivity against citrullinated autoantigens is preceded by reactivity against their native forms [33].

The aim of this study was to assess inflammation of the oral and lung mucosa as a potential cause of RA-AAB production. RA-AAB were assessed in sera of patients without RA with PD, BR or CF. In addition, reactivity against the native forms of citrullinated autoantigens was assessed. RA-AAB serum levels were analyzed within the context of HC and patients with established RA.

## Methods

### Patient groups

Serum autoantibody levels were measured in adult patients without RA with PD) (n = 114), non-CF patients with BR (n = 80) and patients with CF (n = 41). Subjects without systemic disease and without PD served as the HC group (n = 36). Patients with established RA without lung disease and with known periodontal status served as a reference group (RA group, n = 86).

Patients with untreated severe PD were recruited from a referral practice for periodontology (Clinic for Periodontology Groningen). The inclusion criterion was >30 % of sites involved with clinical attachment loss ≥5 mm on the basis of full-mouth oral measurements [34]. The exclusion criteria were antibiotic use <3 months before inclusion and having systemic disease other than PD. To assess the inflammatory burden exerted by the periodontium, the periodontal inflamed surface area (PISA) [35] was quantified.

Sera from patients with BR without CF and without RA from a previously conducted randomized controlled trial were included [36]. Baseline serum samples were used to avoid a possible influence of treatment on antibody levels. Sera from a cohort of patients with CF without RA who visited the Department of Pulmonology of the University Medical Center Groningen for routine checkups were included. For BR and CF patients, the number of exacerbations was based on the number of antibiotic courses received 12 months before inclusion. The percentage predicted forced expiratory volume in 1 second (%FEV_1_) at inclusion was used as a disease activity measure.

HC subjects were recruited from among subjects planned for first consultation at the dental department of the University Medical Center Groningen. Periodontal health was assessed using the Dutch periodontal screening index (DPSI) [37]. An inclusion criterion was DPSI score ≤2 (absence of PD), and exclusion criteria were antibiotic use <3 months before inclusion and presence of systemic disease.

Patients with established RA without lung disease at the time of inclusion and with known periodontal status as assessed by the DPSI served as a reference group for serological measurements. RA disease activity was assessed using the Disease Activity Score 28 tender and swollen joint count (DAS28).

In PD, RA and HC subgingival microbiological samples were tested for presence of *P. gingivalis* by using anaerobic culture techniques (for details, see [38]).

Participants provided written informed consent before study enrollment according to the Declaration of Helsinki. This study was conducted with the approval of the Medical Ethical Committee of the University Medical Center Groningen (METC UMCG 2009/356, METC UMCG 2011/010) and Medical Ethical Committee Noord-Holland (METC Noord-Holland M07-002).

### Laboratory measurements

IgG anti-cyclic citrullinated protein antibody (anti-CCP) levels were measured using a commercial anti-CCP2 kit (Euro Diagnostica, Malmö, Sweden) according to the manufacturer’s protocol. Samples with a value <25 U/ml were measured again with an adjusted protocol in which samples were diluted 1:10 instead of 1:50. The diagnostic cutoff value was defined as >25 U/ml according to the manufacturer’s instruction. However, IgG anti-CCP was not used as a diagnostic test for RA in this study; therefore, seropositivity was defined as >2 SD above the mean of HC (2.2 U/ml), analogous to the other autoantibodies measured.

IgA anti-CCP measurements were performed using a modification of the anti-CCP2 kit (Euro Diagnostica). Sera were diluted 1:50 using the dilution buffer provided by the manufacturer. The secondary antibody was horseradish peroxidase (HRP)-conjugated polyclonal goat anti-human IgA (SouthernBiotech, Birmingham, AL, USA), diluted 1:20,000 in phosphate-buffered saline (PBS) with 1 % bovine serum albumin (BSA) and 0.05 % Tween-20 (Sigma-Aldrich, St. Louis, MO, USA). The color reaction was performed using tetramethylbenzidine (Sigma-Aldrich) and hydrogen peroxide. A pool of four sera from RA patients with high levels of IgA anti-CCP served as a calibrator for the standard curve expressed in arbitrary units per milliliter (AU/ml) and starting at 200 AU/ml. Seropositivity was defined as >2 SD above the mean of HC.

To assess the citrulline-specific nature of the response, sera were tested on plates coated with the arginine-containing counterpart of CCP2: cyclic arginine peptide (CAP, kindly provided by Euro Diagnostica), following almost the same protocol as described for the IgG and IgA CCP2 enzyme-linked immunosorbent assay (ELISA). A dilution of patient serum with high levels of IgG anti- CAP served as a calibrator for the standard curve, starting at 100 AU/ml. Similarly, IgA anti-CAP reactivity was measured with a dilution of a high-level responding serum serving as a standard curve, starting at 100 AU/ml. Seropositivity was defined as >2 SD above the mean of HC.

Levels of IgG anti-CarP antibodies against carbamylated fetal calf serum were assessed using a protocol described by Shi et al. [8]. Seropositivity was defined as >2 SD above the mean of a distinctive HC cohort [8].

The specificity of the response against citrullinated proteins was assessed by testing reactivity against four synthetic citrullinated peptides that are known autoantigens in RA: fibrinogen-1 (Fib1, β-chain amino acids 36–52, NEEGFFSACitGHRPLDKK), fibrinogen-2 (Fib2, β-chain amino acids 60–74, CitPAPPPISGGGYCitACit), α-enolase (CEP-1, KIHACitEIFDSCitGNPTVE) and vimentin (Vim1, VYATCitSSAVCitLCitSSV). Every peptide was linked with its N terminus to biotin with a spacer (SGSG) in between. Reactivity against the citrullinated and native forms of the peptides was measured according to the method of van de Stadt et al. [39] with some modifications. In short, 96-well Costar plates (Corning, Corning, NY, USA) were coated overnight with 5 μg/ml streptavidin (Rockland Immunochemicals, Limerick, PA, USA) in PBS. Subsequently, plates were blocked for at least 1 hour with 2 % BSA and 0.05 % Tween-20 in PBS followed by incubation with the biotin-labeled peptides (0.5 μg/ml in PBS). Next, serum samples were diluted 1:100 in high-performance ELISA buffer (Sanquin, Amsterdam, the Netherlands) and incubated on the plates. Reactivity was detected with HRP-conjugated monoclonal mouse anti-human IgG (clone JDC-10; SouthernBiotech) diluted 1:2000 in PBS with 1 % BSA and 0.05 % Tween-20 or with HRP-conjugated polyclonal goat anti-human IgA (SouthernBiotech) diluted 1:4000 in the same dilution buffer. Bound antibodies were visualized by using tetramethylbenzidine and hydrogen peroxide. Reactivity against a citrullinated peptide and its native counterpart was measured on the same plate. Every serum sample was measured in duplicate, and a positive control serum sample was applied on each plate. The citrulline-specific response was expressed as the difference in optical density (ΔOD) between the citrullinated peptide and its native form, and it was considered positive when ΔOD was >2 SD above the mean of HC. Likewise, the arginine-specific response was expressed as ΔOD between the native peptide and its citrullinated form, and it was considered positive when ΔOD was >2 SD above the mean of HC.

IgM and IgA RF levels were assessed using a validated in-house ELISA [40]. Levels were expressed in international units (IU) per milliliter, and seropositivity was defined as >10 IU/ml for IgM RF and >25 IU/ml for IgA RF [40].

C-reactive protein levels were measured by performing ELISA (DuoSet; R&D Systems, Minneapolis, MN, USA).

Absorbance was read at 450 nm in an EMax microplate reader, and antibody levels were calculated by using SoftMax PRO software (Molecular Devices, Sunnyvale, CA, USA).

### Statistical analysis

Statistical analysis was performed using GraphPad Prism software (version 5.00 for Windows; GraphPad Software, La Jolla, CA, USA) and IBM SPSS Statistics for Windows software (version 20.0; IBM, Armonk, NY, USA). Normality was tested using D’Agostino-Pearson omnibus K2 test. For group comparisons, a Mann–Whitney *U* test was used for continuous variables and Fisher’s exact test for categorical variables. Comparison of RA-AAB levels between the groups was done with Kruskal–Wallis one-way analysis of variance with Dunn’s multiple-comparisons post-test compared with HC if overall *p* < 0.05. A logistic regression model was used to analyze the association of the diseases with seropositivity for anti-CCP and RF. The model was adjusted for age, sex and smoking (former and current versus never). A logistic regression model appeared to be better than a linear regression model owing to non-normally distributed residuals. Associations of anti-CCP, RF and anti-CarP with disease activity were assessed by using a Mann–Whitney *U* test or an unpaired *t*-test with or without Welch’s correction, depending on normality and equality of variances. Correlations between different parameters were assessed by Spearman’s ρ.

## Results

The vast majority of all patient groups was Caucasian. The demographic and clinical characteristics of patient groups are listed in Table 1. Age varied significantly between the groups, being related to the specific ages at which a certain disease is mostly present; for example, patients with CF are of young age owing to the low survival rate of the disease.

**Table 1:** Patient characteristics

Patient group	Rheumatoid arthritis	Periodontitis	Bronchiectasis	Cystic fibrosis	Healthy controls	*p* value (vs. HC)
Subjects (n)	86	114	80	41	36	
Age, yr, median (IQR)	57 (48–64)	50 (45–57)	65 (56–71)	28 (21–36)	26 (24–46)	***RA, PD and BR
Female (%)	56	59	63	49	60	n.s.
Current smoker (%)	60	42	2.5	0	14	**PD, *for BR and CF
Ever smoker (%)	17	36	44	0	8.3	**PD, ***BR
Never smoker (%)	22	22	54	100	78	***PD, *BR, **CF
PISA (cm^2^), median (IQR)	n.a.	14 (9.0–19)	n.a.	n.a.	n.a.	
%FEV_1_, median (IQR)	n.a.	n.a.	81 (60–97)	54 (36–80)	n.a.	
Exacerbations (n), median (IQR)	n.a.	n.a.	4 (3–6)	2 (1–3)	n.a.	
DAS28, median (IQR)	2.2 (1.7–2.8)	n.a.	n.a.	n.a.	n.a.	
CRP (mg/L), median (IQR)	1.9 (1.0–6.0)	1.0 (0.6–2.4)	5 (2.0–13)	6.0 (4.0–14)	0.4 (0.3–1.5)	***RA, BR and CF
No periodontitis (%)	31	0	n.a.	n.a.	100	
Moderate periodontitis (%)	41	0	n.a.	n.a.	0	
Severe periodontitis (%)	28	100	n.a.	n.a.	0	
*Porphyromonas gingivalis*-positive (%)	14	43	n.a.	n.a.	0	
MTX (%)	71					
aTNFα (%)	10					
SASP (%)	3.5					
MTX + aTNFα (%)	3.5					
MTX + SASP (%)	4.7					
Other (%)	3.5					
None (%)	3.5					

*aTNFα* anti-TNFα inhibitors, *CRP* C-reactive protein, *DAS28* Disease Activity Score 28 tender and swollen joint count, *Exacerbations* based on the number of antibiotic courses 12 months before inclusion, *%FEV*_*1*_ percentage predicted forced expiratory volume, *MTX* methotrexate, *n.a.* not assessed, *n.s.* not significant, *PISA* periodontal inflamed surface area, *SASP* sulfasalazine

*p < 0.05, **p < 0.01, ***p < 0.0001 (Kruskal–Wallis one-way analysis of variance with Dunn’s multiple-comparisons post-test or Fisher’s exact test with two-tailed *p* value)

### Anti-CCP and anti-CAP levels

**Fig. 1:**
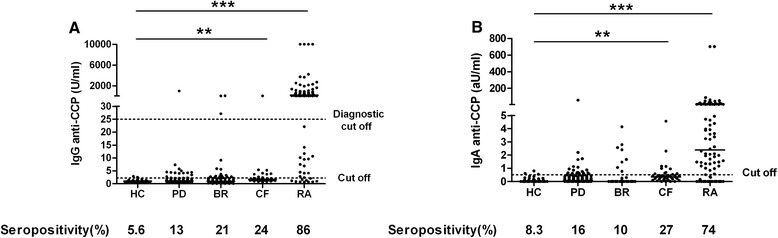
Serum immunoglobulin G (IgG) (**a**) and IgA anti-cyclic citrullinated peptide (anti-CCP) (**b**) levels in healthy controls (HC) and in patients with periodontitis (PD), bronchiectasis (BR), cystic fibrosis (CF) and rheumatoid arthritis (RA). Cutoff values are indicated: diagnostic cutoff (25 U/ml) and >2 SD above the mean of HC for IgG anti-CCP and >2 SD above the mean of HC for IgA anti-CCP. Seropositivity (%) is indicated for cutoff based on >2 SD above the mean of HC. Bar indicates the median. **p < 0.01, ***p < 0.0001, Kruskal–Wallis one-way analysis of variance with Dunn’s multiple-comparisons post-test compared with HC if overall *p* < 0.05

**Fig. 2:**
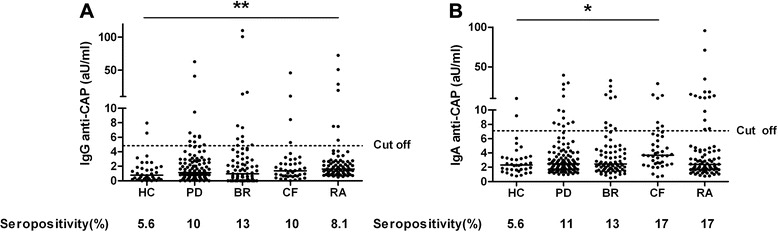
Serum immunoglobulin G (IgG) (**a**) and IgA anti-cyclic arginine peptide (anti-CAP) (**b**) levels in healthy controls (HC) and in patients with periodontitis (PD), bronchiectasis (BR), cystic fibrosis (CF) and rheumatoid arthritis (RA). CAP represents the native counterpart of CCP. Cutoff values are indicated: >2 SD above the mean of HC. Bar indicates the median. *p < 0.05, **p < 0.01, Kruskal–Wallis one-way analysis of variance with Dunn’s multiple-comparisons post-test compared with HC if overall *p* < 0.05

Compared with HC, IgG and IgA anti-CCP levels were increased in patients with CF (both *p* < 0.01) and patients with RA (both *p* < 0.0001). IgG anti-CCP seropositivity was 13 %, 21 %, 24 % and 86 % in PD, BR, CF and RA patients, respectively, and 5.6 % in HC. According to the diagnostic cutoff, IgG anti-CCP seropositivity was 0.9 %, 3.8 %, 2.4 % and 76 % in PD, BR, CF and RA, respectively, and absent in HC. Seropositivity for IgA anti-CCP was 16 %, 10 %, 27 % and 74 % in PD, BR, CF and RA patients, respectively, and 8.3 % in HC (Fig. 1). Reactivity against the native counterpart of CCP (anti-CAP) was, compared with HC, increased in RA patients for IgG anti-CAP (*p* < 0.01) and in CF patients for IgA anti-CAP (*p* < 0.05), although this was not necessarily reflected in increased seropositivity (Fig. 2). Correlations for IgG anti-CCP and IgG anti-CAP levels were found in HC (ρ = 0.57, *p* < 0.001), patients with PD (ρ = 0.32, *p* < 0.001) and patients with BR (ρ = 0.47, *p* < 0.0001), and a trend was observed in patients with CF (ρ = 0.28, *p* = 0.08). IgA anti-CCP and IgA anti-CAP levels were correlated in HC (ρ = 0.41, *p* < 0.05), patients with PD (ρ = 0.39, *p* < 0.0001), patients with CF (ρ = 0.38, *p* < 0.05) and patients with RA (ρ = 0.21, *p* < 0.05).

### Rheumatoid factor

**Fig. 3:**
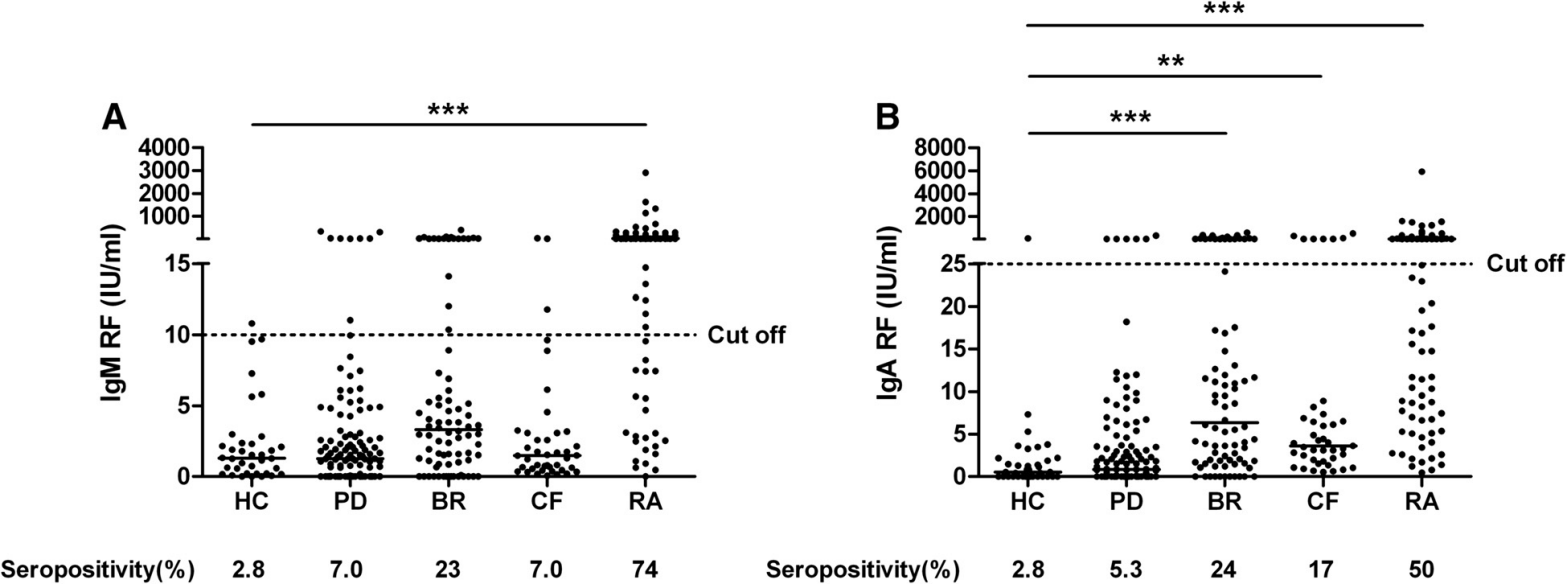
Serum immunoglobulin M rheumatoid factor (IgM RF) (**a**) and IgA RF levels (**b**) in healthy controls (HC) and in patients with periodontitis (PD), bronchiectasis (BR), cystic fibrosis (CF) and rheumatoid arthritis (RA). Cutoff values are indicated: 10 IU/ml for IgM RF and 25 IU/ml for IgA RF. Bar indicates the median. **p < 0.01, ***p < 0.0001, Kruskal–Wallis one-way analysis of variance with Dunn’s multiple-comparisons post-test compared with HC if overall *p* < 0.05

Compared with HC, IgM RF levels were increased in patients with RA only (p < 0.0001), while IgA RF levels were increased in BR (p < 0.0001), CF (p < 0.01) and RA patients (p < 0.0001). IgM RF seropositivity was 7 %, 23 %, 7 % and 74 % in PD, BR, CF and RA patients respectively, and 2.8 % in HC. Seropositivity for IgA RF was 5.3 %, 24 %, 17 %, 50 % in PD, BR, CF and RA patients respectively, and 2.8 % in HC (Fig. 3).

### Anti-CarP antibodies and peptide specific reactivity

Compared with HC, IgG anti-CarP levels were increased in patients with RA (*p* < 0.0001). Seropositivity for IgG anti-CarP was observed in PD, BR and CF patients (3.5 %, 3.8 % and 7.3 %, respectively), but not in HC (Table 2). The percentage of seropositive patients with RA (48 %) was congruent with anti-CarP seropositivity in another Dutch cohort of patients with RA (45 %) [41]. Compared with that in HC, IgG reactivity against all investigated citrullinated peptides was increased in patients with RA (*p* < 0.0001). With the exception of patients with RA, IgG seropositivity against the various citrullinated peptides was not increased in other patient groups studied (Table 2). Compared with that in HC, IgA reactivity against citrullinated fibrinogen-1 was increased in patients with RA (*p* < 0.01), as was seropositivity (19 %) (Table 2).

**Table 2:** Percentages of seropositivity for anti-carbamylated antibodies and various citrullinated peptides and their native arginine counterparts according to cutoff levels of >2 SD above the mean of healthy controls

Patient group	Healthy controls	Periodontitis	Bronchiectasis	Cystic fibrosis	Rheumatoid arthritis
Anti-CarP IgG (% pos.)	0	3.5	3.8	7.3	48
Peptides IgG (% pos.)					
Cit. fibrinogen-1	2.8	0.9	1.3	0	55
*Arg. fibrinogen-1*	0	0.9	1.3	2.4	0
Cit. fibrinogen-2	0	4.4	2.5	0	71
*Arg. fibrinogen-2*	2.8	3.5	3.8	4.9	2.3
Cit. α-enolase	0	0.9	2.5	0	38
*Arg. α-enolase*	2.8	1.8	6.3	4.9	1.2
Cit. vimentin	0	1.8	0	0	48
*Arg. vimentin*	5.6	5.3	1.3	7.3	1.2
Peptide IgA (% pos.)					
Cit. fibrinogen-1	0	0.9	0	0	8.1
*Arg. fibrinogen-1*	2.8	0.9	2.5	0	0
Cit. fibrinogen-2	2.8	2.6	0	0	19
*Arg. fibrinogen-2*	0	7.9	2.5	9.8	4.7
Cit. α-enolase	0	2.6	0	0	7.0
*Arg. α-enolase*	8.3	2.6	6.3	7.3	4.7
Cit. vimentin	0	0	0	0	4.7
*Arg. vimentin*	5.6	4.4	2.5	4.9	2.3

*Arg.* Arginine, *Anti-CarP* anti-carbamylated protein; *Cit.* citrulline, *Ig* immunoglobulin, *% pos.* percentage positive

Compared with that of HC, IgA reactivity against native fibrinogen-2 (*p* < 0.0001) and vimentin (*p* < 0.01) was increased in patients with CF. No differences in seropositivity were observed for the various native peptides between all groups for both immunoglobulin isotypes (Table 2).

### Regression analysis

Logistic regression analysis, adjusted for age, sex and smoking (former and current versus never), revealed that IgG anti-CCP seropositivity was more frequent in BR (odds ratio [OR], 8.6; 95 % CI, 1.5–50; *p* < 0.05) and RA (OR, 226; 95 % CI, 39–1309; *p* < 0.0001). The association with PD was borderline significant (OR, 5.2; 95 % CI, 0.99–27; *p* = 0.05). IgA anti-CCP seropositivity was associated with CF (OR, 4.4; 95 % CI, 1.1–18; *p* < 0.05) and RA (OR, 43; 95 % CI, 10–187; *p* < 0.0001). IgM RF seropositivity was associated with RA (OR, 10; 95 % CI, 10–757; *p* < 0.0001), whereas the association with BR was borderline significant (OR, 9.1; 95 % CI, 1–84; *p* = 0.05). IgA RF seropositivity was associated with CF (OR, 10; 95 % CI, 1.1–97; *p* < 0.05) and RA (OR, 20; 95 % CI, 2.4–163; *p* < 0.01). Smoking was only of influence on the association of IgA RF with RA (*p* < 0.01), as there were no smokers among the patients with CF. Apart from that, smoking, age and sex had no influence on the association of the diseases with anti-CCP or RF seropositivity.

### Association of disease activity with autoantibody status

Pulmonary function, measured as %FEV_1_, was significantly worse in BR and CF patients seropositive for IgA RF (*p* < 0.01 and *p* < 0.05, respectively). Disease activity, as measured by the number of exacerbations 12 months before inclusion based on the number of antibiotic courses, was associated with seropositivity for IgA anti-CCP in patients with CF (*p* < 0.01). Disease extent of PD, measured as PISA, was negatively associated with anti-CarP seropositivity (*p* < 0.05). RA disease activity, as measured by DAS28, was associated with seropositivity for IgG anti-CCP (*p* < 0.05) and IgA RF (*p* < 0.05), whereas the association with anti-CarP was borderline significant (*p* = 0.05).

## Discussion

To our knowledge, this is the first study in which serum IgA anti-CCP and anti-CarP levels have been assessed in non-RA patients with inflammation of oral or lung mucosal tissues. Both IgG and IgA anti-CCP levels were increased in patients with CF compared with HC, and IgA anti-CCP seropositivity was associated with presence of CF.

Besides one study reporting 1 of 45 adult patients with CF seropositive for IgG anti-CCP [23], no data on anti-CCP levels in patients with CF have been published. IgG anti-CCP seropositivity was associated with presence of BR, whereas the association with PD was borderline significant. According to the diagnostic cutoff for IgG anti-CCP, similar seropositivity (3.3 %) has been reported in a comparable BR patient cohort [42], whereas seropositivity of up to 8 % has been reported in patients with PD [43–45]. However, these studies comprised PD patient groups of limited sample size compared with our PD group. Our results for patients with PD are better than those reported by de Pablo et al. [33], who found, according to their diagnostic cutoff, 1 % IgG anti-CCP seropositivity in patients with PD.

Both IgM and IgA RF were increased in patients with CF compared with HC, and IgA RF seropositivity was associated with presence of CF. The former findings are in accord with other reports in the literature [24]; however, Koch et al. [23] found only slightly elevated RF in a cohort of 65 adult and pediatric CF patients and concluded that these laboratory findings were mostly nonspecific. IgM RF was increased in patients with BR compared with HC, and the association of IgM RF seropositivity with presence of BR was borderline significant. Seropositivity for IgM RF in BR (23 %) is in accord with a recent comparable study [42]. The importance of RF and anti-CCP in BR and CF patients is emphasized by the fact that pulmonary function was significantly worse in BR and CF patients seropositive for IgA RF, which has been reported for patients with CF [46]. In addition, the number of exacerbations in patients with CF was associated with IgA anti-CCP seropositivity.

Anti-CarP seropositivity was rather specific for RA. The importance of anti-CarP was underlined by a correlation with RA disease activity, in accordance with the findings of Shi et al. [8]. Recently, next to citrullination, carbamylation has been hypothesized to play a role in the association of PD and RA [30]. Serum anti-CarP levels were not increased in patients with PD, although seropositivity in HC was absent. The extent of periodontal disease was not associated with anti-CCP or RF seropositivity; nevertheless, the unknown periodontal status of BR and CF patients remains a limitation of our study. The extent of periodontal disease was negatively associated with anti-CarP seropositivity, the implication of which is unclear.

Our HC should ideally be better age-matched, because a trend toward increased amounts of serum IgM RF with advancing age has been described [47], especially in advanced elderly people without RA (aged >78 years) [48]. However, the latter study found that only 1 of 300 advanced elderly subjects was IgG anti-CCP-seropositive when the cutoff level was set at the 98th percentile of blood donors aged 40–65 years (mean age, 50 years), which represents ages of patients for whom immunological tests for RA are typically performed [48]. This age range is comparable to that of our PD, BR and RA patients. Together with the absence of significant influence of age in the logistic regression model, we assume that RF and anti-CCP seropositivity is not much influenced by age differences among patient groups. Regarding anti-CarP, we cannot comment on the contribution of the age factor in our patient groups, because its relationship with age has not been tested in healthy populations.

Among the PD, BR and CF patients and HC, no differences were found in IgA or IgG seropositivity for the citrullinated peptides of candidate autoantigens in RA (e.g., citrullinated α-enolase). Increased IgG reactivity against citrullinated α-enolase in patients with PD was reported previously [33, 45]. Of note, these studies showed increased reactivity against the native peptide of citrullinated α-enolase as well. Therefore, the observed increased levels of anti-citrullinated α-enolase were probably, at least in part, not citrulline-specific. To rule out this possibility, we assessed the difference in reactivity against the citrullinated peptide and its native counterpart. Likewise, the specific reactivity against the native forms of the peptides was assessed. Break of tolerance toward native forms of citrullinated autoantigens may lead to reactivity against citrullinated autoantigens via epitope spreading [33]. Brink et al. [32] supported this hypothesis by reporting IgG seropositivity for various native peptides in a limited number of pre-symptomatic patients with RA. The correlations between anti-CCP and anti-CAP levels in our patient groups suggest that at least part of the observed anti-CCP reactivity is not citrulline-specific. A non-citrulline-specific anti-CCP response has been reported in patients with tuberculosis [49, 50]. In addition, in all our patient groups, there was limited IgA and IgG seropositivity toward one or more native peptides. Especially the patients with CF showed an increased IgA response against CAP, native fibrinogen-2 and vimentin peptides, but this not reflected in increased seropositivity for these antigens. Seropositivity for native antigens was also observed in HC and might not be of clinical relevance. It remains unclear whether reactivity toward citrullinated peptides can be preceded by reactivity against their native forms, because no longitudinal data of our study subjects were available.

*P. gingivalis* has been speculated to contribute to the initiation of ACPA generation because of PPAD expression. No increased reactivity was found against citrullinated α-enolase in patients with PD, the candidate RA autoantigen that shows sequence similarity with *P. gingivalis* enolase [51]. In contrast to Lappin et al. [45], in our study we found no differences in ACPA levels between patients with PD with or without subgingival *P. gingivalis* (data not shown). Differences in study methodology, including *P. gingivalis* detection, could have contributed to this different study result.

Low serum anti-CCP levels have also been reported for gastrointestinal mucosal inflammation [52, 53]. Because RA-AAB are thought to be induced locally, serum levels might not necessarily reflect local autoantibody production. ACPA have been found in gingival crevicular fluid of patients with PD [54] and in sputum of subjects at risk for RA [22]. To our knowledge, local ACPA production in the gastrointestinal tract has not yet been investigated.

## Conclusions

Although overall levels were low, the presence of IgG and IgA anti-CCP and IgM and IgA RF is independent of age, sex and smoking associated with lung mucosal inflammation (BR and CF) and may be associated with oral mucosal inflammation (PD). RA-AAB in the peripheral blood in the presence of mucosal inflammation, albeit not according to the diagnostic cutoff level, supports the hypothesis that formation of these autoantibodies may be induced at inflamed mucosal surfaces. To further determine whether mucosal inflammation functions as a site for induction of RA-AAB and precedes RA, longitudinal studies are necessary in which RA-AAB of specifically the IgA isotype should be assessed in inflamed mucosal tissues and/or in their inflammatory exudates.

## Abbreviations

ACPA: anti-citrullinated protein antibody
AU: arbitrary units
anti-CAP: anti-cyclic arginine peptide
anti-CarP: anti-carbamylated protein
anti-CCP: anti-cyclic citrullinated peptide
BR: bronchiectasis
BSA: bovine serum albumin
CAP: cyclic arginine peptide
CF: cystic fibrosis
CI: confidence interval
CRP: C-reactive protein
DAS28: Disease Activity Score 28 tender and swollen joint count
DPSI: Dutch periodontal screening index
ELISA: enzyme-linked immunosorbent assay
%FEV_1_: percentage predicted forced expiratory volume in 1 second
HC: healthy controls
HRP: horseradish peroxidase
Ig: immunoglobulin
IQR: interquartile range
IU: international units
MTX: methotrexate
OD: optical density
OR: odds ratio
PAD: peptidyl arginine deiminase
PBS: phosphate-buffered saline
PD: periodontitis
PISA: periodontal inflamed surface area
PPAD: *Porphyromonas gingivalis* peptidyl arginine deiminase
RA: rheumatoid arthritis, RA-AAB, rheumatoid arthritis–associated autoantibodies
RF: rheumatoid factor
SASP: sulfasalazine
SD: Standard deviation
aTNFα: anti-tumor necrosis factor α inhibitors
